# Shared and differential default-mode related patterns of activity in an autobiographical, a self-referential and an attentional task

**DOI:** 10.1371/journal.pone.0209376

**Published:** 2019-01-04

**Authors:** Paola Fuentes-Claramonte, Marta Martín-Subero, Pilar Salgado-Pineda, Silvia Alonso-Lana, Ana Moreno-Alcázar, Isabel Argila-Plaza, Aniol Santo-Angles, Anton Albajes-Eizagirre, Maria Anguera-Camós, Antoni Capdevila, Salvador Sarró, Peter J. McKenna, Edith Pomarol-Clotet, Raymond Salvador

**Affiliations:** 1 FIDMAG Germanes Hospitalàries Research Foundation, Barcelona, Spain; 2 CIBERSAM (Centro de Investigación Biomédica en Red de Salud Mental), Barcelona, Spain; 3 Universitat Autònoma de Barcelona, Barcelona, Spain; 4 Institut de Neuropsiquiatria i Addiccions, Centre Fòrum Research Unit, Parc de Salut Mar, Barcelona, Spain; 5 IMIM (Hospital del Mar Medical Research Institute), Barcelona, Spain; 6 Radiology Unit, Hospital de la Santa Creu i Sant Pau (HSCSP), Barcelona, Spain; 7 CIBER-BBN (Centro de Investigación Biomédica en Red en Bioingeniería, Biomateriales y Nanomedicina), Barcelona, Spain; University of California, San Francisco, UNITED STATES

## Abstract

The default-mode network (DMN) comprises a set of brain regions that show deactivations during performance of attentionally demanding tasks, but also activation during certain processes including recall of autobiographical memories and processing information about oneself, among others. However, the DMN is not activated in a homogeneous manner during performance of such tasks, so it is not clear to what extent its activation patterns correspond to deactivation patterns seen during attention-demanding tasks. In this fMRI study we compared patterns of activation in response to an autobiographical memory task to those observed in a self/other-reflection task, and compared both to deactivations observed during the n-back working memory task. Autobiographical recall and self-reflection activated several common DMN areas, which were also deactivated below baseline levels by the n-back task. Activation in the medial temporal lobe was seen during autobiographical recall but not the self/other task, and right angular gyrus activity was specifically linked to other-reflection. ROI analysis showed that most, but not all DMN regions were activated above baseline levels during the autobiographical memory and self-reflection tasks. Our results provide evidence for the usefulness of the autobiographical memory task to study DMN activity and support the notion of interacting subsystems within this network.

## Introduction

The default mode network (DMN) is a set of brain regions that typically show synchronized activity in a variety of behavioral states including resting states but also tasks with different cognitive requirements [1–4]. Its core regions include the medial prefrontal cortex (mPFC), the posterior cingulate cortex (PCC)/precuneus, and the angular gyrus in the inferior parietal lobule. Parts of the temporal lobe, including its neocortex and the hippocampus and parahippocampus, are often also considered part of the network [5,6] while some specific portions of the ventrolateral (VLPFC) and dorsolateral prefrontal cortex (DLPFC), usually extending from the mPFC, have been included as well [7,8]. A considerable amount of research about the DMN has been focused on its deactivation during the performance of tasks demanding externally oriented attention (i.e. greater activity at rest or fixation than during task execution, [3,5,9,10]) that led to its initial identification as a “task-negative” network [1]. However, later work has shown its involvement in several cognitive functions [10], and its specific role is presently uncertain, with proposals ranging from low-level ‘background’ monitoring of the environment to internal mentation detached from the outside world, construction of mental simulations or maintenance of the self-concept [5,11]. Changes in DMN activity have also been linked to attentional fluctuations during task performance [12–14].

Highly relevant to theories of DMN function is the finding that, while many cognitive tasks produce deactivation in the DMN, others increase its activity in one or more of its parts [10]. One such task is autobiographical recall, i.e. the conscious recollection of relatively rich images from a person’s past, typically evoked experimentally by a cue word or phrase. A meta-analysis of PET and fMRI studies of autobiographical recall by Svoboda et al. [15] found activations corresponding quite closely to several regions of the DMN, including the medial frontal cortex, the PCC/precuneus, the inferior parietal cortex and the hippocampus. Other studies have shown similar activation patterns [16–22]. Convergent evidence from lesion and EEG studies also highlight the involvement of DMN areas in autobiographical memory [23,24].

The DMN is also engaged in tasks that, similar to autobiographical memory, share the need to process self-relevant information, including self-reflection, making social and emotional judgments about oneself and others, envisioning the future or performing theory of mind operations [5,6,17,22]. However, despite the similarity between DMN regions and regions activated by self-reference tasks, the two are not identical as there are brain areas that also respond differentially, for example portions of the ventral mPFC or the precuneus [20,25–27]. Similarly, although it has not been studied yet, it is quite likely that the extent of the overlap between regions activated in self referential tasks and regions deactivated by standard cognitive tasks is also incomplete. More detailed consideration of the brain activations that take place in response to self-reference and other tasks which activate the DMN might also be desirable because the control conditions used in such tasks are often attention-demanding (e.g., semantic processing, arithmetic tasks), and so seem capable of producing DMN deactivation. In such circumstances it becomes unclear whether activations seen in DMN areas will be a result of activation over baseline levels in the self-relevant conditions, or rather reflect deactivation of these same regions in the control conditions, or a combination of both [3]. For example, Holt et al. [28] found significant differences in the mPFC between a self-reflection and an affect-labeling condition. However, these differences arose from a deactivation in the affect-labeling condition rather than activation in self-reflection: in the latter, activity was close to the baseline level (fixation). A similar result was found in Jenkins & Mitchell [29] for the same region. Detailed examination of the activation and deactivation patterns in different DMN regions is needed to clarify this question. In this sense, note that baseline conditions in most cognitive tasks consist of rest or fixation periods, which are examples of relatively unconstrained and low-demanding tasks against which activation or deactivation is tested. Thus, the terms ‘baseline’ and ‘rest’ are herein used to refer to those low-demanding tasks, during which several cognitive processes are probably still taking place.

Another aspect that warrants further exploration in the study of the DMN is the heterogeneity found within the network: some authors propose that the DMN is organized into subsystems linked to specific roles that interact with each other: a medial temporal lobe (hippocampal) subsystem associated with autobiographical and episodic memory and a dorsal mPFC subsystem, also including the lateral temporal and inferior parietal cortex, linked to mentalizing and social processing [5,26,27,30]. This hypothesis stems from the observation that the dorsal mPFC and the medial temporal lobes do not show functional connectivity with each other but are both connected to core DMN regions, which would serve as hubs within the network, coordinating the associated subsystems [5,31]. Recent findings have shown that DMN subsystems can be identified by functional connectivity in individual brains, which have supported the existence of two subnetworks within the DMN, only one of which is functionally coupled with the hippocampal formation [32]. Such findings highlight the importance of studying the patterns of activation of the different DMN regions in response to different task conditions. Previous studies that have directly compared to what extent different self-relevant tasks overlap or differ in brain activation have found support for these two subsystems, with the medial temporal lobe linked to episodic memory, future planning and prospection, while the dorsal subsystem has been associated to mentalizing, making self-referential decisions, and theory of mind [22,26,31,33]. These studies also showed that midline structures were commonly activated in all self-referential tasks. Less studied has been, however, the degree of overlap between the activation of DMN areas in self-reference tasks and their deactivation in classical attention-demanding paradigms.

In the present study, we aim to (1) validate an autobiographical memory task as a suitable paradigm for studies targeting the DMN, and (2) examine activations and deactivations during performance of this autobiographical memory task and a self/other reflection task. We will compare these with an attention-demanding task, the n-back paradigm, which has been consistently found to produce deactivation in the DMN [34–36]. We expect that midline DMN regions (mPFC, PCC) will be activated in autobiographical memory and self reflection, but deactivated in the n-back task. We also expect the hippocampus to be preferentially activated for autobiographical memory and the inferior parietal cortex to be preferentially activated for self-reflection, while both will be deactivated in the n-back. Activity in these regions will be analyzed to explore whether it corresponds to activations or deactivations with respect to baseline levels which consist of fixation periods. An important concern that arises in studies about the DMN is that memory or self-reflection processes might be engaged not only during the task, but also during fixation, which may prevent the observation of differences between them [15]. For this reason, our study will use two different analysis approaches: first, a conventional whole-brain analysis comparing each condition of interest with a matched control condition that involves similar perceptual and linguistic demands; and second, a ROI analysis that tests activation levels against a fixation baseline. This will allow us to identify the brain regions involved in each task and examine which conditions drive activation and deactivation in the DMN.

## Materials and methods

### Participants

The sample consisted of 37 healthy volunteers recruited from the community. Exclusion criteria included left-handedness, neurological illness, present or past diagnosis of psychiatric disorder, first-degree relatives with a psychiatric diagnosis, alcohol or substance abuse or dependence (excluding nicotine) in the last year, head trauma with loss of consciousness, and general exclusion criteria for MRI such as presence of metals within the body or pregnancy. One participant was excluded due to anatomical abnormality found in the MRI scan, leaving 36 to be included in the analyses. Two participants were excluded from the autobiographical memory task and 3 participants were excluded from the self-reflection task due to excessive head movement. Demographic characteristics of the final samples used for the three tasks are shown in Table 1.

**Table 1:** Demographic characteristics of the final samples for each task.

	Autobiographical memory task	Self-reflection task	N-back task
**Total N**	34	33	36
**Mean age (SD)**	41.97 (11.63)	41.67 (11.67)	41.19 (11.99)
**Age range**	23–65	23–65	18–65
**Gender**	19 M / 15 F	19 M / 14 F	20 M / 16 F

All participants gave written informed consent prior to participation in accordance to the Declaration of Helsinki. All the study procedures had been previously approved by the Clinical Research Ethics Committee of the Sisters Hospitallers (Comité de Ética de Investigación Clínica de las Hermanas Hospitalarias). Participants received a gift-card as a compensation for their participation in the study.

### Experimental tasks

#### Autobiographical memory task

The task used in the present work was designed using as a reference the paradigm applied by Oertel-Knöchel et al. [21] which was modified to allow direct comparison between a condition where an autobiographical memory was evoked, and an otherwise similar control condition where no memory was evoked. Immediately before the fMRI session, each participant was interviewed by one of the researchers to obtain autobiographical memory-evoking and non-evoking stimuli. For this, they were administered prompts from the Autobiographical Memory Interview (AMI) [37], which take the form of phrases, and the Crovitz test [38], which take the form of words. During the interview, participants were required to provide memories of specific incidents from their past based on these prompts; between four and six memories for each time period were provided: childhood (until age 11), adolescence (age 12–18), adulthood (age 19 and above) and recent events (the last year). For each memory, a pair of two words was constructed in agreement with the participant, able to evoke its associated memory upon presentation. This interview lasted approximately 1 hour. The stimuli finally used in the fMRI paradigm consisted of groups of three words personalized for each participant. The first word referred to one of four possible time periods (“childhood”, “adolescence”, “adult” and “recently”), and the other two words corresponded to one of the pairs that had evoked autobiographical memories in the prior testing (e.g. “childhood grandmother cake”, “adult car robbery”). Following the scoring system of the AMI, to be included as stimuli, memories were required to be given the maximum score of 3, based on the descriptive richness of the account of the incident and its specificity in time and place.

For the control condition, we generated similar groups of three words for each participant that did not correspond to any of the retrieved memories and for which no association with a specific memory was reported. This control condition differs from Oertel-Knöchel et al.’s [21] original paradigm, where the control condition was a semantic completion task. It was chosen to be similar to the autobiographical condition at the perceptual and linguistic levels (e.g. semantic word processing), but unable to evoke personal memories.

The paradigm was a block design with 10 blocks of control stimuli alternated with 10 blocks of autobiographical stimuli; all blocks lasted 20s. Each block contained two word groups of control and autobiographical stimuli from the same time period, lasting 10s each. Between blocks, a fixation cross was presented during 16s providing the baseline (total baseline time = 5 min and 4 s). Participants were instructed to silently read the words and to recall the memory associated with them if there was one.

#### Self-reflection task

This task was adapted from the one described in Modinos et al. [39] and consisted of a personalized self-other judgment task. Before scanning, participants were asked to choose an acquaintance to think about inside the scanner. The chosen individual had to be familiar to the participant but not too close to avoid eliciting strong feelings towards them (for example, a classmate or a co-worker).

During the task, participants viewed a series of affirmations about themselves (self), an acquaintance (other), or about general knowledge (facts)–the last was the control condition. They had to respond with a button press indicating whether they considered the sentence to be true or false. In the self condition, the sentences referred to personal qualities, attributes or attitudes, such as “In general, I like order” or “I am a tense or very nervous person”. Similarly, in the other condition sentences referred to the personality traits and behavior of the chosen acquaintance. Examples of sentences here were “OTHER often makes decisions without thinking” or “OTHER usually has very good ideas” (OTHER being replaced in the task by the chosen person’s name). In the facts condition, sentences referred to general knowledge such as “A decade is a period of ten years” or “Insects only have four legs”. In the self and other conditions half of the sentences had a positive valence and half had a negative valence, while in the facts condition half of the sentences were true and the other half were false.

The task consisted of 54 trials (18 per condition) arranged in a block design. Each block started with an instruction screen indicating the condition type (“Sentences about me”, “Sentences about OTHER” and “Sentences about facts”), which lasted 3s. After a 1s delay, three trials were presented, each lasting 9s, where the sentence appeared in the center of the screen and the options “Yes” and “No” appeared at the bottom-right and bottom-left corners, respectively, to act as a reminder of the required response (“Yes” with the right index finger, and “No” with the left index finger). Trials were separated by a 1s blank screen. After three trials, the next block started, with a total of 6 blocks per condition. Every 3 blocks there was a baseline period of 16s in which a fixation cross was presented (total baseline time = 1 min 20 s). Block order was pseudorandomized, with each of the three conditions occurring once between baseline periods.

#### N-back task

The task consisted of two levels of memory load (1-back and 2-back) presented in a blocked design manner; in the 1-back condition, participants had to respond with a key press when the letter shown on the screen was the same as the one that was presented immediately before, whereas in the 2-back condition they had to respond when the letter was the same as that presented two letters previously. Each block consisted of 24 letters which were shown every two seconds (1 second on, 1 second off) and all blocks contained 5 repetitions (1-back and 2-back depending on the block) located randomly within the block. In order to identify which task had to be performed, letters were shown in green in the 1-back blocks and in red in the 2-back blocks. Four 1-back and four 2-back blocks were presented in an interleaved way, and between them, a baseline stimulus (an asterisk flashing with the same frequency as the letters) was presented for 16 seconds (total baseline time = 1 min 52 s). All individuals went through a training session before entering the scanner.

All tasks were administered in the same fMRI session and in the same order (n-back, autobiographical memory and self-reflection) to ensure the procedure was identical for all participants. During the fMRI session, participants were asked whether they had been attending the task and stimuli after each task. The three experimental tasks were programmed with the Tcl programming language and executed in an Alienware (Alienware Corporation, Miami, Florida, USA) laptop running on Windows 10. In the scanner, stimuli were presented on VisualSystem goggles mounted on the head coil, and responses were made and registered with the MRI-compatible response device Response-grip. Stimulus presentation was synchronized with the scanner through a SyncBox (VisualSystem goggles, Response-grip and SyncBox are manufactured by NordicNeurolab, AS, Bergen, Norway).

### Image acquisition

Images were acquired with a 3T Philips Achieva scanner (Philips Medical Systems, Best, The Netherlands). Functional data were acquired using a T2*-weighted echo-planar imaging (EPI) sequence with the following acquisition parameters: TR = 2000ms, TE = 30ms, Flip angle = 78^O^, in-plane resolution = 3 × 3mm, FOV = 240mm, slice thickness = 3mm, inter-slice gap = 1mm. The autobiographical memory task consisted of 370 volumes, the self-reflection task consisted of 364 volumes, and the n-back task consisted of 266 volumes. Slices (32 per volume) were acquired with an ascending order parallel to the AC-PC plane. The first 10 volumes were discarded to avoid T1 saturation effects. Before the functional sequences, a high-resolution anatomical 3D volume was acquired using a TFE (Turbo Field Echo) sequence for anatomical reference and inspection (TR = 8.15ms; TE = 3.73ms; Flip angle = 8^O^; voxel size = 0.9375 × 0.9375mm; slice thickness = 1mm; slice number = 160; FOV = 240mm).

### Image preprocessing and analysis

Preprocessing and analyses were carried out with the FEAT module included in the FSL (FMRIB Software Library) software [40]. Preprocessing was identical for the three paradigms and included motion correction (using the MCFLIRT algorithm with 6 degrees of freedom) and co-registration and normalization to a common stereotactic space (Montreal Neurological Institute template with 2 × 2 × 2mm resolution) using linear transformations with 12 degrees of freedom. Before group analyses, normalized images were spatially filtered with a Gaussian filter (FWHM = 5mm). Individuals with an estimated maximum absolute movement >3.0mm or an average absolute movement >0.3mm were excluded from analyses to minimize unwanted movement-related effects.

Statistical analyses were performed by means of General Linear Models (GLMs) designed independently for each task. For the autobiographical memory task two regressors of interest were defined at the single-subject level analysis (memory blocks vs. control blocks) and the GLM was fitted to generate activation maps of each condition compared to baseline and for the comparison between conditions. For the n-back task, the same procedure was applied, defining the two task conditions as regressors of interest (1-back and 2-back). Given our interest in the deactivation of DMN regions in this task, we specifically compared the 2-back condition with baseline. For the self-reflection task, three regressors of interest were defined in the GLMs corresponding to the three task conditions (self, other, facts). Instruction screens were modeled through an additional nuisance regressor. GLMs were fitted to generate activation maps for each of the three conditions of interest compared to baseline and for the comparisons between conditions (self vs. facts, other vs. facts, self vs. other).

For the three models, temporal derivatives for each regressor of interest, as well as movement parameters (six in total, three rotations and three translations) were also included as additional regressors. Fixation periods were not modeled and thus acted as implicit baseline (i.e. to compare a condition of interest of any given task with its baseline periods, the average BOLD signal from all the baseline periods across the whole task is subtracted from that of the blocks corresponding to the condition of interest). Images were high-pass filtered with a 130s cutoff. At the group level, activations and deactivations were assessed with one-sample t-tests on the contrasts defined at the subject level with mixed-effects models [41]. Statistical tests were carried out at the cluster level with a corrected *p* < 0.05 using Gaussian random field methods and considering a gray matter mask to restrict analyses to gray matter areas. A threshold of *z* = 3.1 at the voxel level was used to define the initial set of clusters.

Apart from obtaining maps of significant activations for each task, we performed region of interest (ROI) analyses to examine the activation and deactivation patterns of relevant DMN regions individually and across tasks. To define the ROIs, we used the Neurosynth package [42] to retrieve an automated meta-analysis of 516 studies using the term “default mode” (see http://neurosynth.org/analyses/terms/default%20mode/ for the original meta-analysis map). This meta-analysis provided a reverse-inference map of brain regions preferentially linked with the default mode network, FDR-corrected at *p*<0.001. The map was smoothed with a Gaussian kernel (sigma = 3), thresholded with a minimum *z* value of 1.5, and binarized, to obtain the set of ROIs that were finally included in the analysis: mPFC, PCC/precuneus, right and left angular gyri, right and left medial temporal lobes, and right and left inferior temporal cortex. From each ROI we extracted average parameter estimates for each task condition compared to its corresponding baseline. This allowed us to compare the activation or deactivation levels of relevant DMN regions and to check if the differences between conditions in BOLD response found in the whole-brain analyses were mainly due to activation in the condition of interest, to deactivation in the control condition, or both.

## Results

### Autobiographical memory task

**Fig 1:**
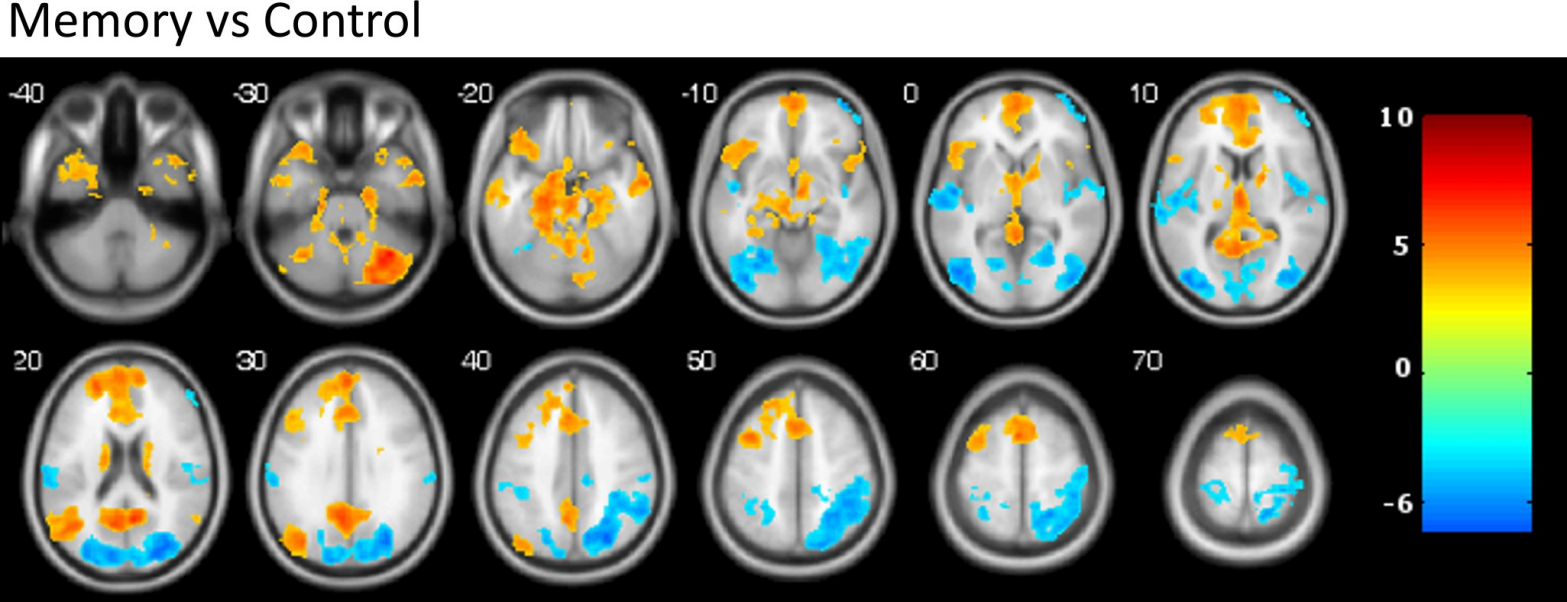
Activation map for the autobiographical memory task. Warm colors represent memory > control contrast, cold colors represent control > memory contrast. Numbers indicate z coordinate in MNI space. Images are displayed in neurological convention (right is right). Color bar depicts Z values.

In the autobiographical memory condition, compared to the control condition, the participants showed extensive activation in the mPFC and PCC, the left angular gyrus, and the medial temporal lobe bilaterally, including hippocampus and parahippocampus. Other regions showing increased activity in the memory condition were the VLPFC and DLPFC, the temporal poles and the cerebellum (see Table 2 and Fig 1). Regions showing greater activity in the control than in the autobiographical memory condition included areas of the occipital and parietal cortices, superior temporal cortex and right frontal pole.

**Table 2:** Significant activation in the autobiographical memory task.

Region	Hemisphere	x	y	z	Z-value	Cluster size	Sig.
**Memory > Control**							
Cerebellum	R	36	-62	-30	7.08	14853	*p*<0.001
Precuneus	R	8	-56	20	6.35		
PCC	L	-6	-52	34	6.12		
Hippocampus	L	-18	-14	-22	5.67		
	R	24	-20	-16	5.18		
Caudate	R	18	2	14	5.41		
Frontal pole	L	-24	52	18	5.78	8759	*p*<0.001
SMA	L	-2	10	60	5.58		
Medial prefrontal cortex	L	-2	64	4	5.56		
DLPFC	L	-38	10	52	5.49		
Angular gyrus/Middle occipital	L	-46	-76	34	5.53	1317	*p*<0.001
Insula	R	48	12	-8	4.66	200	*p* = 0.007
**Control > Memory**							
Occipital cortex	R	26	-72	38	6.1	18370	*p*<0.001
	L	-44	-82	8	5.9		
Fusiform gyrus	L	-34	-54	-16	5.25		
	R	36	-60	-14	4.91		
Lingual gyrus	L	-22	-64	-10	5.22		
Cuneus	L	-14	-90	24	5.7		
Superior temporal cortex	L	-50	-10	0	5.2		
Inferior parietal cortex	R	54	-52	36	4.99		
Angular gyrus	R	30	-54	46	5.26		
Superior parietal	R	20	-56	60	5.11		
Superior occipital cortex	R	26	-72	38	6.11		
Middle frontal gyrus	R	42	60	-8	5.12	301	*p*<0.001

Coordinates are shown in MNI space. Cluster size shows the number of voxels. R: Right; L: Left; PCC: Posterior cingulate cortex; SMA: Supplementary motor area, DLPFC: Dorsolateral prefrontal cortex.

### Self-reflection task

**Fig 2:**
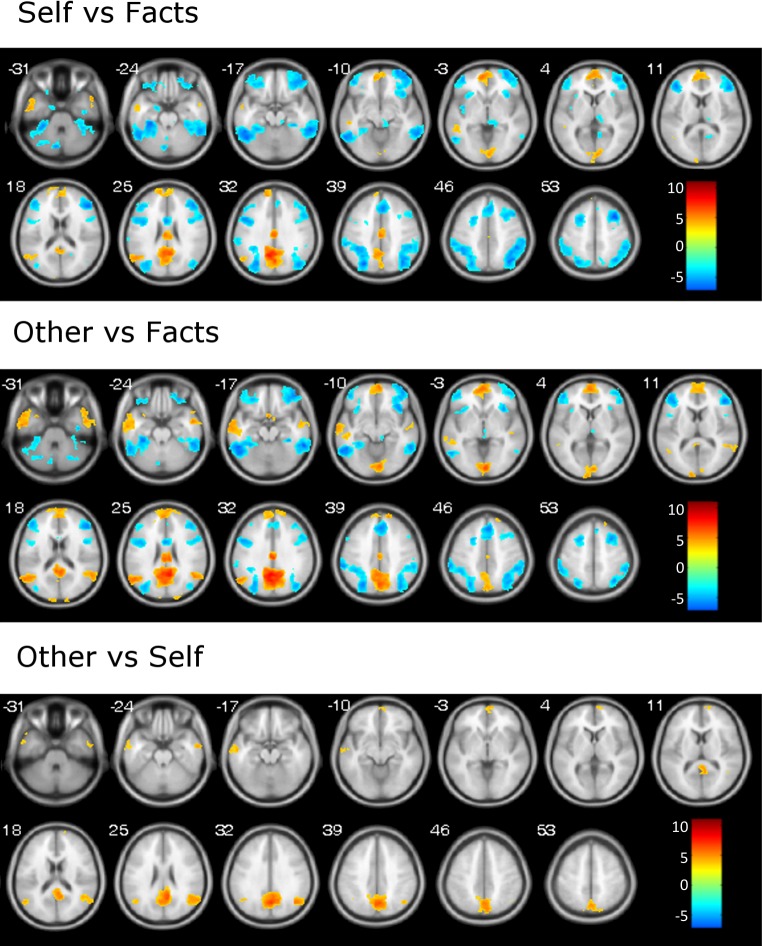
Activation map for the self-reflection task. Warm colors represent self > facts (upper row), other > facts (middle row) and other > self (lower row) contrasts, cold colors represent facts > self (upper row) and facts > other (middle row) contrasts. Numbers indicate z coordinate in MNI space. Images are displayed in neurological convention (right is right). Color bar depicts Z values.

Contrasts revealed broadly similar activation maps for the self and other conditions relative to the facts condition. Both activated the mPFC and frontal pole, the left angular gyrus, the bilateral middle temporal gyrus and temporal poles, the mid-cingulate cortex, the PCC, the precuneus and the calcarine cortex. The other condition, but not the self condition, also activated the right angular gyrus (Table 3, Fig 2).

**Table 3:** Significant activation in the self-reflection task.

Region	Hemisphere	x	y	z	Z-value	Cluster size	Sig.
**Self > Facts**							
Precuneus/PCC	L	-8	-58	32	7.53	2111	*p*<0.001
Medial prefrontal cortex/Frontal pole	L	-8	58	-6	5.62	1745	*p*<0.001
Calcarine cortex	B	0	-90	-4	4.81	569	*p*<0.001
Middle/posterior cingulate cortex	B	0	-22	40	5.62	530	*p*<0.001
Angular/Middle temporal gyrus	L	-52	-62	24	5.01	508	*p*<0.001
Temporal pole	R	46	-4	-38	5.33	483	*p*<0.001
	L	-48	6	-40	4.67	479	*p*<0.001
Middle temporal cortex	L	-52	-36	-4	4.05	179	*p* = 0.006
**Facts > Self**							
DLPFC	R	34	14	54	7.34	5763	*p*<0.001
Occipital cortex	L	-28	-74	40	6.78	3602	*p*<0.001
Angular gyrus	R	42	-66	44	6.77	3237	*p*<0.001
Fusiform gyrus/Inf. Temporal cortex	L	-32	-34	-26	6.72	3027	*p*<0.001
	R	60	-46	-14	6.7	2303	*p*<0.001
Inferior frontal gyrus	L	-42	40	12	6.05	2052	*p*<0.001
		-48	8	30	5.86	739	*p*<0.001
Medial superior frontal cortex	R	4	32	40	6.11	909	*p*<0.001
DLPFC	L	-24	10	58	5.62	687	*p*<0.001
Cerebellum	L	-8	-78	-28	5.77	421	*p*<0.001
Thalamus	R	10	-20	8	4.57	417	*p*<0.001
Precuneus	R	8	-64	68	4.17	217	*p* = 0.002
Fusiform gyrus	L	-30	-8	-38	5.15	180	*p* = 0.005
Insula	L	-32	20	-2	4.36	146	*p* = 0.016
		-42	0	0	4.93	133	*p* = 0.025
ACC	L	-2	6	26	5.86	138	*p* = 0.021
Lingual gyrus	R	8	-52	6	4.19	124	*p* = 0.033
**Other > Facts**							
Precuneus/PCC	L	-4	-52	30	8.33	4072	*p*<0.001
Medial prefrontal cortex	B	0	60	0	5.51	2551	*p*<0.001
Inferior temporal cortex	L	-56	-8	-28	5.87	2106	*p*<0.001
Temporal pole	R	48	-4	-36	5.87	1276	*p*<0.001
		4	-86	-6	6.63	1104	*p*<0.001
Middle temporal/Angular gyrus	L	-52	-62	22	5.98	820	*p*<0.001
Angular gyrus	R	46	-56	24	5.24	620	*p*<0.001
**Facts > Other**							
Inferior temporal cortex/Fusiform gyrus	L	-52	-50	-18	6.83	2992	*p*<0.001
Lateral orbitofrontal cortex	R	44	54	-12	7.04	2938	*p*<0.001
Inferior parietal cortex/supramarginal	L	-62	-38	44	6.67	2693	*p*<0.001
	R	34	-78	38	5.93	2411	*p*<0.001
DLPFC/Inferior frontal gyrus	L	-44	46	6	6	1756	*p*<0.001
Inferior temporal cortex/Fusiform gyrus	R	60	-46	-18	7.34	1666	*p*<0.001
DLPFC	R	30	14	54	6.1	1474	*p*<0.001
Medial superior frontal cortex	R	2	30	44	6.32	1164	*p*<0.001
Inferior frontal gyrus	L	-46	8	30	5.88	754	*p*<0.001
Superior frontal cortex	L	-22	14	50	6.07	505	*p*<0.001
Insula	L	-32	24	-8	4.88	224	*p* = 0.001
Fusiform gyrus	R	40	-14	-30	4.91	176	*p* = 0.005
	L	-30	-8	-40	4.83	114	*p* = 0.038
Cerebellum	R	26	-68	-32	4.68	150	*p* = 0.011
	L	-8	-78	-28	4.54	130	*p* = 0.022
Thalamus	R	2	-18	0	4.1	148	*p* = 0.012
ACC	B	0	6	26	5.65	122	*p* = 0.028
**Other > Self**							
Precuneus/PCC	R	2	-64	32	6.57	3326	*p*<0.001
Angular gyrus	R	44	-64	32	5.18	858	*p*<0.001
Temporal pole/Middle temporal gyrus	L	-40	16	-36	4.47	510	*p*<0.001
Angular/Middle temporal gyrus	L	-54	-64	18	3.99	268	*p*<0.001
Medial prefrontal cortex	R	4	68	-4	4.21	254	*p*<0.001
Middle/Inferior temporal gyrus	R	58	-6	-26	4.33	117	*p* = 0.025

Coordinates are shown in MNI space. Cluster size shows the number of voxels. PCC: Posterior Cingulate Cortex; ACC: Anterior Cingulate Cortex; DLPFC: Dorsolateral prefrontal cortex; R: Right; L: Left; B: Both.

The facts condition was associated with increased activity with respect to both the self and other conditions in a set of areas that included the lateral frontal cortex bilaterally and the medial superior frontal cortex. Bilateral activity was also seen in the superior parietal cortex extending to the inferior parietal area as well as in the insula, the fusiform gyrus, and the thalamus.

While no region showed increased activity in the self compared to the other condition, several brain areas were more active in the other than in the self condition. These included the precuneus and PCC, the left and right angular gyri, the middle temporal cortex, the right frontal pole and both temporal poles.

### N-back task

**Fig 3:**
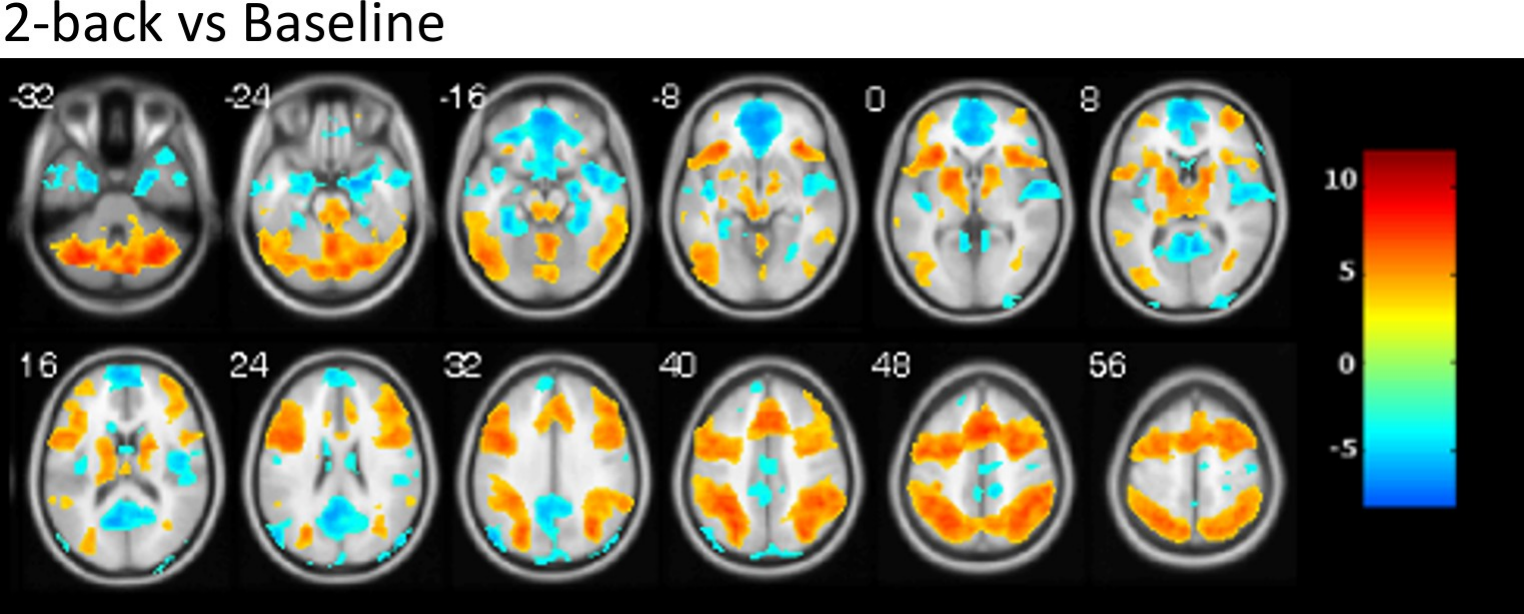
Activation map for the n-back task. Warm colors show activation of the 2-back condition compared to baseline, cold colors show deactivation. Numbers indicate *z* coordinate in MNI space. Images are displayed in neurological convention (right is right). Color bar depicts Z values.

Because in this and in previous studies by our group (e.g. [34]), activations and deactivations were considerably more pronounced in the 2-back vs. baseline than in the 1-back vs. baseline contrast, the analyses reported here are focused in the 2-back condition (activations and deactivations in the 1-back condition were highly similar but with lower intensity). Activation in the 2-back condition compared to baseline was seen in a large cluster involving left and right lateral prefrontal cortices, the anterior cingulate cortex (ACC), lateral and medial parietal cortices, the occipital cortex, the basal ganglia and the thalamus. Deactivation (i.e. baseline > 2-back) was found in the mPFC, the medial temporal lobe including the hippocampus and extending into the temporal poles (bilaterally), the superior temporal gyrus and the posterior insula, and PCC/precuneus (see Table 4 and Fig 3).

**Table 4:** Significant activation in the n-back task.

Region	Hemisphere	x	y	z	Z-value	Cluster size	Sig.
**2-back > Baseline**							
Cerebellum	R	30	-68	-30	8.26	45930	*p*<0.001
	L	-30	-66	32	7.93		
Dorsal ACC	R	2	16	48	7.82		
Inferior temporal cortex	R	58	-46	-12	5.17		
Inferior frontal cortex	R	34	28	-6	7.15		
	L	-32	24	-6	6.84		
DLPFC/Superior frontal cortex	R	36	52	10	6.15		
Inferior parietal cortex	R	56	-40	42	6.68		
Middle occipital cortex	R	30	-70	36	7.54		
Putamen	L	-16	6	2	6.45		
**Baseline > 2-back**							
Medial prefrontal cortex	R	4	52	-10	7.11	11747	*p*<0.001
Precuneus/PCC	L	-6	-56	22	6.56	4212	*p*<0.001
Hippocampus	L	-22	0	-26	6.33	3428	*p*<0.001
Angular gyrus	L	-50	-76	30	6.19	374	*p*<0.001
Mid-cingulate cortex	R	2	-16	48	4.22	323	*p*<0.001
	R	10	-36	48	4.78	173	*p* = 0.010
Postcentral gyrus	R	48	-20	58	4.14	187	*p* = 0.007
Precentral gyrus	R	24	-28	74	4.64	150	*p* = 0.020

Coordinates are shown in MNI space. Cluster size shows the number of voxels. PCC: Posterior Cingulate Cortex; ACC: Anterior Cingulate Cortex; DLPFC: Dorsolateral prefrontal cortex. R: Right; L: Left.

### ROI analyses

**Fig 4:**
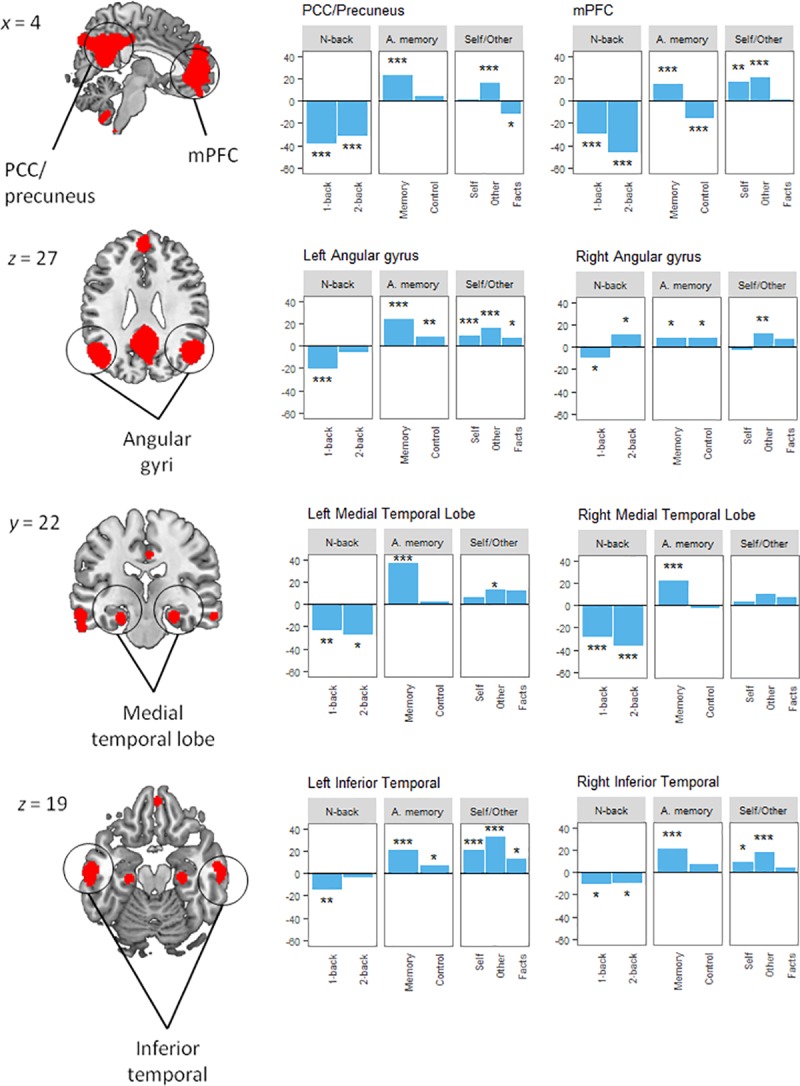
Activation in DMN regions in ROI analysis. Vertical axis shows parameter estimates. *p<0.05, **p<0.01, ***p<0.001(the last being equivalent to a Bonferroni corrected p<0.05).

For each region, we tested whether its activation was different from baseline levels with a one-sample t-test in each task condition. Only those participants with valid data for the three tasks were included in the ROI analyses (n = 33). Results are reported in Table 5 and Fig 4. A Bonferroni corrected *p* value of 0.001 (= 0.05/56 tests performed) was considered to control for false positives. The PCC/precuneus ROI was activated in the autobiographical and other-reflection conditions, but not in the self condition. Oppositely, it was deactivated in the 2-back, and also slightly deactivated in the facts condition (although at an uncorrected significance threshold). Similarly, the mPFC was activated by autobiographical memory and other-reflection, and more weakly by self-reflection (only at uncorrected level), while it was deactivated in 2-back and in the control condition from the autobiographical memory task.

**Table 5:** Significant activations for the ROI analyses.

	N-back				Autobiographical memory				Self/Other reflection					
	1-back		2-back		Memory		Control		Self		Other		Facts	
Region	t_(32)_	p	t_(32)_	p	t_(32)_	p	t_(32)_	p	t_(32)_	p	t_(32)_	p	t_(32)_	p
**PCC**	**-8.150**	**<0.001**	**-5.911**	**<0.001**	**5.444**	**<0.001**	1.014	0.318	0.396	0.695	**3.924**	**<0.001**	-2.457	0.02
**mPFC**	**-5.118**	**<0.001**	**-6.99**	**<0.001**	**3.478**	**0.001**	**-3.715**	**0.001**	3.047	0.005	**3.889**	**<0.001**	0.22	0.828
**Left Angular**	**-4.999**	**<0.001**	-1.044	0.304	**7.45**	**<0.001**	2.834	0.008	**4.166**	**<0.001**	**5.478**	**<0.001**	2.252	0.031
**Right Angular**	-2.235	0.033	2.563	0.015	2.458	0.02	2.612	0.014	-0.332	0.742	2.941	0.006	1.367	0.181
**Left MTL**	-3.044	0.005	-2.673	0.012	**6.668**	**<0.001**	0.501	0.62	0.929	0.36	2.181	0.037	1.844	0.075
**Right MTL**	**-5.689**	**<0.001**	**-6.328**	**<0.001**	**5.895**	**<0.001**	-0.536	0.595	0.483	0.632	1.707	0.098	1.234	0.226
**Left Inf. Temp.**	-2.873	0.007	-0.608	0.547	**5.484**	**<0.001**	2.211	0.034	**3.885**	**<0.001**	**5.873**	**<0.001**	2.735	0.01
**Right Inf. Temp.**	-2.498	0.018	-2.095	0.044	**4.463**	**<0.001**	-0.342	0.734	2.112	0.043	**3.882**	**<0.001**	0.793	0.434

Bold shows significant activations surviving multiple comparison correction. PCC: Posterior Cingulate Cortex; mPFC: medial Prefrontal Cortex; MTL: Medial temporal lobe; Temp: Temporal.

The angular gyrus showed a different profile: the left angular was significantly active in all self-relevant conditions (autobiographical, self-reflection and other-reflection) but was not deactivated in any condition. Its right homologue did not show any significant activation or deactivation at corrected levels, although it was active at an uncorrected level in other-reflection.

Both medial temporal lobes (MTL) were significantly active in the autobiographical memory condition, but not in self/other-reflection. The right MTL was also deactivated in the 2-back. The inferior temporal cortices were both active for autobiographical memory and self/other-reflection (the right temporal only at an uncorrected level for self-reflection), but they were not deactivated in the 2-back.

Finally, we explored if any of the ROIs was preferentially activated by autobiographical memory in contrast with self-reflection. In each ROI, we compared the parameter estimates for the two conditions using a paired samples t-test. We found that activation levels were similar in the mPFC and the temporal poles. However, the MTLs (especially the left), the PCC/precuneus and the left angular gyri were more active in autobiographical memory than in self-reflection, with differences at trend level for the right angular (Table 6).

**Table 6:** Comparison between activation levels in autobiographical memory and self-reflection conditions.

Region	Mean PE Autobigraphical memory	Mean PE Self-reflection	t(32)	p
**PCC**	22.639	1.553	**3.743**	**0.001**
**mPFC**	15.452	16.931	-0.231	0.819
**Left Angular**	23.719	8.917	**4.069**	**<0.001**
**Right Angular**	8.290	-1.264	2.053	0.048
**Left MTL**	36.541	5.908	**3.763**	**0.001**
**Right MTL**	21.642	2.796	3.406	0.002
**Left Inf. Temp.**	20.692	20.839	-0.023	0.981
**Right Inf. Temp.**	16.282	9.099	1.601	0.119

Bold shows significant activations surviving multiple comparison correction. PE: parameter estimates, MTL: Medial temporal lobe.

### Overlap in activation maps

**Fig 5:**
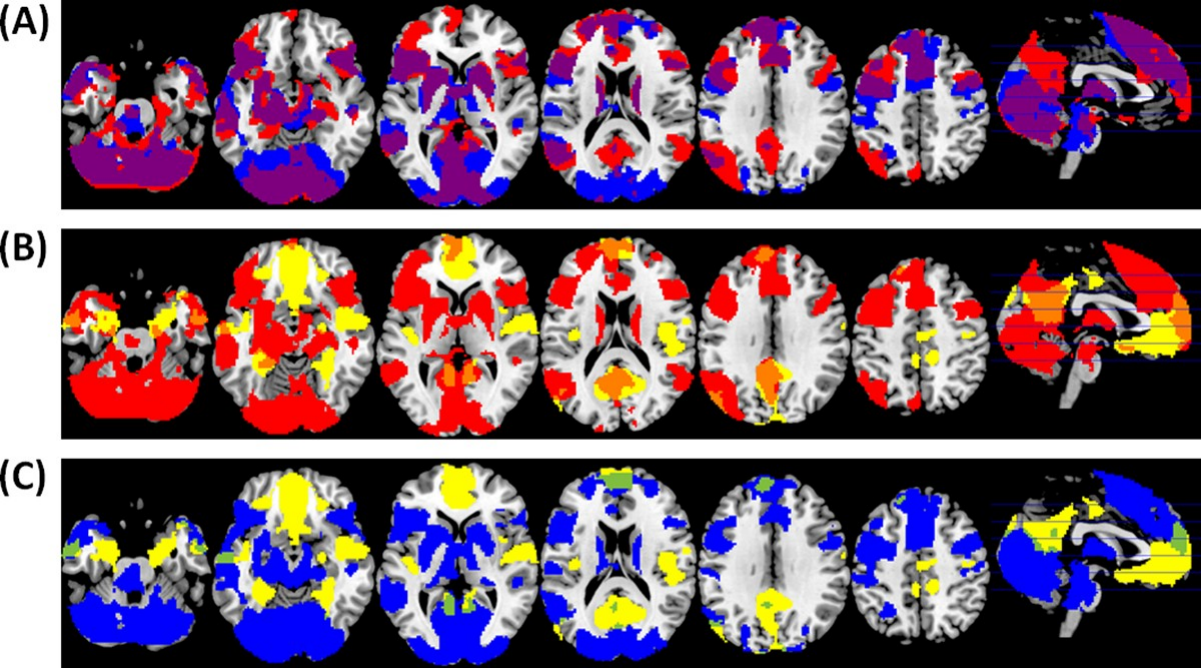
Overlap between activated areas in autobiographical memory and self-reflection, and deactivated areas in 2-back, compared to fixation. (A) Activation maps for autobiographical memory (red) and self-reflection (blue). Overlap is shown in purple. (B) Activation maps for autobiographical memory (red) and 2-back deactivation (yellow). Overlap is shown in orange. (C) Activation maps for self-reflection (blue) and 2-back deactivation (yellow). Overlap is shown in green.

As an alternative way to further explore the similarities and differences between brain areas activated by autobiographical memory and self-reflection, and deactivated by the n-back task, we examined the degree of overlap in the activation maps for each task compared to the low-level baseline (fixation). Results are displayed in Fig 5.

First we overlapped the activation maps corresponding to autobiographical memory and self-reflection vs. fixation (Fig 5A). Considerable overlap was found in the dorsomedial prefrontal cortex, left angular gyrus and temporal lobes, and also outside DMN regions such as the lateral prefrontal cortex, basal ganglia, thalamus and occipital cortex. Consistent with the ROI analysis, the precuneus was activated by autobiographical memory but not self-reflection.

Fig 5B depicts the overlap between autobiographical memory and deactivated areas in 2-back. Overlap is observed in the precuneus, temporal lobes and left angular gyrus. There is also overlap in the medial prefrontal cortex, however in this region deactivation in 2-back seems to be located more ventrally than activation for autobiographical memory.

Activation for self-reflection and deactivation for 2-back showed the least amount of overlap (Fig 5C). It included part of the medial prefrontal cortex (although again activation in self-reflection was more dorsal than deactivation in 2-back), a small cluster in the precuneus, left angular gyrus and temporal poles.

## Discussion

The aim of this study was to analyze the activation patterns of DMN regions during the performance of an autobiographical memory task and a self-reflection task, which both activate DMN areas, and to compare these with the deactivation pattern in a conventional attention-demanding task, the n-back task. It was found that core midline DMN regions (mPFC and PCC) were activated by autobiographical memory and self-reflection but deactivated by the n-back task. We also found evidence for a MTL-hippocampus DMN subsystem preferentially linked to autobiographical memory. Our autobiographical memory task proved to be a simple and useful tool to study DMN activity relative to this process.

Current theoretical approaches to DMN function highlight its involvement in active mental states, contrary to previous accounts that considered it to be a “task-negative” network, and consider that its main function is to support self-generated thought, in contrast with other types of mental activity that are more directed towards external stimuli [5,25,30]. The present results support this view of the DMN, as it is engaged in the processing of self-relevant information and inhibited when directing attention to a cognitive task that does not contain any personal information: individually, both self-relevant tasks activated the regions classically associated to the DMN, and these same regions were deactivated in the n-back task. This pattern was confirmed by the activation maps of the whole-brain analysis and also by the ROI analysis. Moreover, all DMN regions identified in the ROI analyses showed activation above baseline levels for autobiographical memory (some of them also for self/other reflection), which indicates that even if these regions are active during baseline or resting states (e.g. because of mind-wandering during rest periods), they activate further during tasks that involve processing self-relevant information. The ROI analysis also revealed that all the activations seen in the autobiographical > control contrast were genuine activations (i.e. activation above baseline level), and not the result of a deactivation in the control condition.

The core regions (PCC and mPFC), as expected, were active in both the autobiographical memory and the self-reflection tasks. The main difference was observed in the hippocampus and MTL, active for autobiographical memory but not self-reflection. This concurs with the proposal of Buckner et al. [5] that the hippocampus is part of a DMN subsystem linked to autobiographical recall and not to other types of self-referential processing, and with recent findings of a DMN subnetwork functionally coupled with the hippocampal formation [32]. However, we found less evidence for the second DMN subsystem (also proposed in [5]), which was hypothesized to comprise the inferior parietal cortex and to show a preferential involvement in self-reflection, as this region was significantly active for both autobiographical memory and self-reflection in the left hemisphere, while neither of them activated the right. The description of DMN subsystems has also linked the dorsal portion of the mPFC to this second subsystem, while the ventral portion corresponds to the core DMN [27]. Interestingly, our analysis of activation map overlap did show that autobiographical memory and self-reflection activated a more dorsal portion of the mPFC while deactivation in n-back was located more ventrally, although with an area of overlap between both. However, activation in dorsal mPFC was very similar in both DMN-activating tasks. Differences between mPFC subregions might therefore be more related to the need to deactivate in attention demanding tasks than to the specific self-referential process at play. As also shown by the ROI analysis, it is likely that activation in the mPFC in DMN-activating tasks arises not only from activation of this region above baseline levels in the conditions of interest but also from a deactivation in the control conditions as they usually involve some kind of cognitive processing that is not self-generated thought [3].

A novel contribution of the present study is the direct comparison of the two self-relevant tasks with the n-back, a classic cognitive task known to induce DMN deactivation [34–36]. As hypothesized, the n-back task deactivated all the DMN regions that showed increased activity in the self-relevant tasks: mPFC, PCC, left inferior parietal cortex, and MTL. It also deactivated the inferior temporal cortex, a region that has also been linked to the DMN as shown by the Neurosynth automated meta-analysis (see Methods section) and that seems to be involved in semantic processing [43]. From our results, it seems that the DMN exhibits modularity in its activation, with at least one clear subsystem involving the MTL and hippocampus, but it is deactivated as a whole when performing tasks that demand paying attention to external stimuli, although the ROI analysis indicates that deactivation is most pronounced in the core DMN regions. One question that is still a matter of debate is the meaning of these deactivations. Early work proposed that there is a baseline level of activity in the human brain, in which different cognitive processes (e.g. environment monitoring, stimulus-independent thought) take place [3]. Under this perspective, task-induced deactivation occurs when these baseline processes are suspended to perform a task that demands focused attention [9]. Recent accounts have proposed that the DMN has a role on attentional fluctuations, with deactivation occurring when the task is performed in an effortful, controlled way, but not when performance is more automatic [12,13]. Our ROI analysis shows that deactivation was clear in the 1-back (requiring sustained attention) and 2-back (requiring attention and working memory) conditions, especially in the core DMN. However, it was also possible that we had additionally observed deactivation in the control conditions of the other two tasks (reading neutral words and answering questions about the world), but this was limited or absent. While these control tasks also required goal-directed cognition, their attentional demands were far lower than those in the n-back. Thus, our results favor an interpretation of DMN deactivation as reflecting interplay between brain networks when effortful cognitive control needs to be displayed. Thus, DMN deactivation might not be only dependent on the particular task, but also on the individual’s performance strategies.

The self/other task provides an additional comparison that, although secondary to the aims of the present work, is also worth considering, namely the direct comparison between self and other-reflection. Compared with the facts condition, that mostly involves semantic processing, the self and other conditions activated roughly the same brain regions (mPFC, PCC, left angular). Direct comparison of self and other revealed that these regions were more active for other than for self-reflection, which also activated the right angular gyrus. These findings contrast with previous evidence of a distinction between the ventral and dorsal portions of the mPFC for self and other-reflection, respectively [44]. A possible explanation is that self and other judgments required similar, overlapping cognitive processes in our task, given that reflection about personality and behavior of others should rely in the recall of personal interactions with the other individual. In contrast, some of the previous studies that found greater mPFC in self rather than other-reflection used a public person (e.g. a politician) as “other” [29,45]. Reflecting upon such a public figure, who is not personally known by the participant, might in fact involve semantic processing instead of self-reflection or autobiographical memory–resulting in increased mPFC activation in the self > other contrast. However, this question warrants further study because other previous works have found ACC and mPFC activity in self > other contrasts using tasks very similar to ours [39,46].

The PCC was significantly activated in other, but not in self-reflection. Greater activity in the PCC in the other rather than in the self condition had been previously reported with a very similar task [39,47,48], and this result had also been explained by the need of engaging in autobiographical memory (linked to the PCC) during other-reflection, presumably to retrieve previous interactions with the individual to make a judgment [49]. Our results also support this view, as autobiographical memory but not self-reflection significantly engaged the PCC. In the case of the angular gyrus, especially in the right hemisphere, this region is part of the temporo-parietal junction, which has been proposed as a candidate for the processes of self/other differentiation based on its role in multisensory integration, action imitation and mentalizing [50,51]. A role in self/other differentiation aligns well with our result of right angular gyrus activation in other but not in self-reflection.

### Limitations

Although we directly compared the autobiographical memory and self-reflection tasks in the ROI analysis, this result should be interpreted with caution given that parameter estimates come from two different paradigms. While alternatively it would have been possible to design a single task with multiple experimental conditions, using different paradigms allowed us to compare each condition of interest with a well-matched control condition and avoid the processes engaged in one task interfering in the other. On the other hand, results from the whole-brain analysis do not exactly match those from the ROI analysis, probably because the regions in each of them do not overlap completely. However, the use of independently defined ROIs was necessary to perform ROI statistics and avoid “double dipping” [52]. The two types of analyses can be seen as complementary and taking them both into account facilitates the interpretation of the results. Finally, although participants were asked about their engagement during the tasks in the periods without behavioral output, their degree of attention or engagement could not be objectively assessed. In the future, the use of eye-tracking devices during fMRI exploration could overcome this limitation.

### Conclusions

The present study supports the current theoretical views of DMN function as the basis for a type of cognition that is self-generated and, even though it can be elicited by external stimuli, it is directed towards the self and personally relevant information. Our results provide evidence of the dynamics of this network: when attention is “internally oriented” (i.e. directed towards the self or towards self-related information), activity increases, and this increase overcomes even the levels of activity found in low-demanding or baseline periods, where DMN activity can also occur as a result of spontaneous mind-wandering. On the contrary, when attention is “externally oriented” (i.e. directed towards a goal-directed task that does not involve the self, such as the n-back task), this network is inhibited and its activation decreases below the resting baseline levels, while the activity of other cognitive networks rises [27]. Our study also supports the existence of task-related modularity within the DMN, with at least one subsystem preferentially linked to autobiographical memory and consisting of medial temporal areas. Future studies may combine different self-referential conditions in a single paradigm to allow more direct comparisons. It might also be interesting to use such a combined task to explore changes occurring in clinical populations with known or suspected DMN alteration, hence contributing to a better characterization of their neurobiological abnormalities.

## References

[1] Fox MD, Snyder AZ, Vincent JL, Corbetta M, Van Essen DC, Raichle ME. The human brain is intrinsically organized into dynamic, anticorrelated functional networks. Proc Natl Acad Sci U S A. 2005;102: 9673–8. 10.1073/pnas.0504136102 15976020 PMC1157105

[2] Krienen FM, Thomas Yeo BT, Buckner RL. Reconfigurable task-dependent functional coupling modes cluster around a core functional architecture. Philos Trans R Soc B Biol Sci. 2014;369 10.1098/rstb.2013.0526 25180304 PMC4150301

[3] Gusnard DA, Raichle ME. Searching for a baseline: Functional imaging and the resting human brain. Nat Rev Neurosci. 2001;2: 685–694. Available: 10.1038/35094500 11584306

[4] Shulman GL, Fiez JA, Corbetta M, Buckner RL, Miezin FM, Raichle ME, et al Common blood flow changes across visual tasks: II. Decreases in cerebral cortex. J Cogn Neurosci. 1997;9: 648–663. 10.1162/jocn.1997.9.5.648 23965122

[5] Buckner RL, Andrews-Hanna JR, Schacter DL. The brain’s default network: anatomy, function, and relevance to disease. Ann N Y Acad Sci. 2008;1124: 1–38. 10.1196/annals.1440.011 18400922

[6] Whitfield-Gabrieli S, Ford JM. Default Mode Network Activity and Connectivity in Psychopathology. Annu Rev Clin Psychol Vol 8. 2012;8: 49–75. 10.1146/Annurev-Clinpsy-032511-143049 22224834

[7] Thomas Yeo BT, Krienen FM, Sepulcre J, Sabuncu MR, Lashkari D, Hollinshead M, et al The organization of the human cerebral cortex estimated by intrinsic functional connectivity. J Neurophysiol. 2011;106: 1125–1165. 10.1152/jn.00338.2011 21653723 PMC3174820

[8] Power JD, Cohen AL, Nelson SM, Wig GS, Barnes KA, Church JA, et al Functional Network Organization of the Human Brain. Neuron. Elsevier Inc.; 2011;72: 665–678. 10.1016/j.neuron.2011.09.006 22099467 PMC3222858

[9] Raichle ME, MacLeod AM, Snyder AZ, Powers WJ, Gusnard DA, Shulman GL. A default mode of brain function. Proc Natl Acad Sci U S A. 2001;98: 676–82. 10.1073/pnas.98.2.676 11209064 PMC14647

[10] Spreng RN. The fallacy of a “task-negative” network. Front Psychol. 2012;3: 145 10.3389/fpsyg.2012.00145 22593750 PMC3349953

[11] Gusnard DA. Being a self: Considerations from functional imaging. Conscious Cogn. 2005;14: 679–697. 10.1016/j.concog.2005.04.004 16256372

[12] Esterman M, Noonan SK, Rosenberg M, Degutis J. In the zone or zoning out? Tracking behavioral and neural fluctuations during sustained attention. Cereb cortex. 2013;23: 2712–23. 10.1093/cercor/bhs261 22941724

[13] Kucyi A, Esterman M, Riley CS, Valera EM. Spontaneous default network activity reflects behavioral variability independent of mind-wandering. Proc Natl Acad Sci. 2016;113: 13899–13904. 10.1073/pnas.1611743113 27856733 PMC5137714

[14] Sali AW, Courtney SM, Yantis S. Spontaneous Fluctuations in the Flexible Control of Covert Attention. J Neurosci. 2016;36: 445–454. 10.1523/JNEUROSCI.2323-15.2016 26758836 PMC4710768

[15] Svoboda E, McKinnon MC, Levine B. The functional neuroanatomy of autobiographical memory: A meta-analysis. Neuropsychologia. 2006;44: 2189–2208. 10.1016/j.neuropsychologia.2006.05.023 16806314 PMC1995661

[16] Addis DR, McIntosh AR, Moscovitch M, Crawley AP, McAndrews MP. Characterizing spatial and temporal features of autobiographical memory retrieval networks: a partial least squares approach. Neuroimage. 2004;23: 1460–71. 10.1016/j.neuroimage.2004.08.007 15589110

[17] Addis DR, Wong AT, Schacter DL. Remembering the past and imagining the future: common and distinct neural substrates during event construction and elaboration. Neuropsychologia. 2007;45: 1363–1377. 10.1016/j.neuropsychologia.2006.10.016 17126370 PMC1894691

[18] Gilboa A, Winocur G, Grady CL, Hevenor SJ, Moscovitch M. Remembering our past: Functional neuroanatomy of recollection of recent and very remote personal events. Cereb Cortex. 2004;14: 1214–1225. 10.1093/cercor/bhh082 15166099

[19] Steinvorth S, Corkin S, Halgren E. Ecphory of Autobiographical Memories: an fMRI Study on Recent and Remote Memory Retrieval. Neuroimage. 2006;30: 285–298. 10.1016/j.neuroimage.2005.09.025 16257547 PMC1513614

[20] Bado P, Engel A, de Oliveira-Souza R, Bramati IE, Paiva FF, Basilio R, et al Functional dissociation of ventral frontal and dorsomedial default mode network components during resting state and emotional autobiographical recall. Hum Brain Mapp. 2014;35: 3302–3313. 10.1002/hbm.22403 25050426 PMC4216410

[21] Oertel-Knöchel V, Reinke B, Hornung A, Knöchel C, Matura S, Knopf M, et al Patterns of autobiographical memory in bipolar disorder examined by psychometric and functional neuroimaging methods. J Nerv Ment Dis. 2012;200: 296–304. 10.1097/NMD.0b013e31824ceef7 22456582

[22] Spreng RN, Grady CL. Patterns of brain activity supporting autobiographical memory, prospection, and theory of mind, and their relationship to the default mode network. J Cogn Neurosci. 2010;22: 1112–1123. 10.1162/jocn.2009.21282 19580387

[23] Philippi CL, Tranel D, Duff M, Rudrauf D. Damage to the default mode network disrupts autobiographical memory retrieval. Soc Cogn Affect Neurosci. 2014;10: 318–326. 10.1093/scan/nsu070 24795444 PMC4350487

[24] Knyazev GG, Savostyanov AN, Bocharov A V., Dorosheva EA, Tamozhnikov SS, Saprigyn AE. Oscillatory correlates of autobiographical memory. Int J Psychophysiol. Elsevier B.V.; 2015;95: 322–332. 10.1016/j.ijpsycho.2014.12.006 25523347

[25] Whitfield-Gabrieli S, Moran JM, Nieto-Castañón A, Triantafyllou C, Saxe R, Gabrieli JDE. Associations and dissociations between default and self-reference networks in the human brain. Neuroimage. 2011;55: 225–232. 10.1016/j.neuroimage.2010.11.048 21111832

[26] Andrews-Hanna JR, Saxe R, Yarkoni T. Contributions of episodic retrieval and mentalizing to autobiographical thought: evidence from functional neuroimaging, resting-state connectivity, and fMRI meta- analyses. Neuroimage. 2014;91: 324–335. 10.1016/j.neuroimage.2014.01.032 24486981 PMC4001766

[27] Andrews-Hanna JR, Smallwood J, Spreng RN. The default network and self-generated thought: Component processes, dynamic control, and clinical relevance. Ann N Y Acad Sci. 2014;1316: 29–52. 10.1111/nyas.12360 24502540 PMC4039623

[28] Holt DJ, Cassidy BS, Andrews-Hanna JR, Lee SM, Coombs G, Goff DC, et al An Anterior-to-Posterior Shift in Midline Cortical Activity in Schizophrenia During Self-Reflection. Biol Psychiatry. 2011;69: 415–423. 10.1016/j.biopsych.2010.10.003 21144498 PMC3740539

[29] Jenkins AC, Mitchell JP. Medial prefrontal cortex subserves diverse forms of self-reflection. Soc Neurosci. 2011;6: 211–218. 10.1080/17470919.2010.507948 20711940

[30] Andrews-Hanna JR. The Brain’s Default Network and Its Adaptive Role in Internal Mentation. Neurosci. 2012;18: 251–270. 10.1177/1073858411403316 21677128 PMC3553600

[31] Andrews-Hanna JR, Reidler JS, Sepulcre J, Poulin R, Buckner RL. Functional-Anatomic Fractionation of the Brain’s Default Network. Neuron. Elsevier Ltd; 2010;65: 550–562. 10.1016/j.neuron.2010.02.005 20188659 PMC2848443

[32] Braga RM, Buckner RL. Parallel Interdigitated Distributed Networks within the Individual Estimated by Intrinsic Functional Connectivity. Neuron. Elsevier Inc.; 2017;95: 457–471.e5. 10.1016/j.neuron.2017.06.038 28728026 PMC5519493

[33] Saxe R, Moran JM, Scholz J, Gabrieli J. Overlapping and non-overlapping brain regions for theory of mind and self reflection in individual subjects. Soc Cogn Affect Neurosci. 2006;1: 229–234. 10.1093/scan/nsl034 18985110 PMC2555418

[34] Pomarol-Clotet E, Salvador R, Sarró S, Gomar J, Vila F, Martínez Á, et al Failure to deactivate in the prefrontal cortex in schizophrenia: dysfunction of the default mode network? Psychol Med. 2008;38: 1185–1193. 10.1017/S0033291708003565 18507885

[35] Gordon EM, Stollstorff M, Vaidya CJ. Using Spatial Multiple Regression to Identify Intrinsic Connectivity Networks Involved in Working Memory Performance. Hum Brain Mapp. 2012;33: 1536–1552. 10.1002/hbm.21306 21761505 PMC3374884

[36] Landin-Romero R, McKenna PJ, Salgado-Pineda P, Sarró S, Aguirre C, Sarri C, et al Failure of deactivation in the default mode network: a trait marker for schizophrenia? Psychol Med. 2015;45: 1315–1325. 10.1017/S0033291714002426 25331916

[37] Kopelman MD, Wilson BA, Baddeley AD. The autobiographical memory interview: A new assessment of autobiographical and personal semantic memory in amnesic patients. J Clin Exp Neuropsychol. Routledge; 1989;11: 724–744. 10.1080/01688638908400928 2808661

[38] Crovitz HF, Sciffman H. Frequency of episodic memories as a function of their age. Bull Psychon Soc. 1974;4: 517–518. 10.3758/BF03334277

[39] Modinos G, Ormel J, Aleman A. Activation of anterior insula during self-reflection. PLoS One. 2009;4: e4618 10.1371/journal.pone.0004618 19242539 PMC2643476

[40] Smith SM, Jenkinson M, Woolrich MW, Beckmann CF, Behrens TEJ, Johansen-Berg H, et al Advances in functional and structural MR image analysis and implementation as FSL. Neuroimage. 2004;23: 208–219. 10.1016/j.neuroimage.2004.07.051 15501092

[41] Beckmann CF, Jenkinson M, Smith SM. General multilevel linear modeling for group analysis in FMRI. Neuroimage. 2003; 10.1016/S1053-8119(03)00435-X 14568475

[42] Yarkoni T, Poldrack RA, Nichols TE, Van Essen DC, Wager TD. NeuroSynth: a new platform for large-scale automated synthesis of human functional neuroimaging data. Front Neuroinformatics Conf Abstr 4th INCF Congr Neuroinformatics. 2011; 10.3389/conf.fninf.2011.08.00058 PMC3146590 21706013

[43] Visser M, Jefferies E, Lambon Ralph MA. Semantic processing in the anterior temporal lobes: a meta-analysis of the functional neuroimaging literature. J Cogn Neurosci. 2010;22: 1083–94. 10.1162/jocn.2009.21309 19583477

[44] Denny BT, Kober H, Wager TD, Ochsner KN. A Meta-Analysis of Functional Neuroimaging Studies of Self and Other Judgments Reveals a Spatial Gradient for Mentalizing in Medial Prefrontal Cortex. J Cogn Neurosci. 2012;24: 1742–1752. 10.1162/jocn_a_00233 22452556 PMC3806720

[45] Bergström ZM, Vogelsang DA, Benoit RG, Simons JS. Reflections of Oneself: Neurocognitive Evidence for Dissociable Forms of Self-Referential Recollection. Cereb Cortex. 2014;25: 2648–2657. 10.1093/cercor/bhu063 24700584 PMC4537426

[46] Modinos G, Mechelli A, Ormel J, Groenewold NA, Aleman A, McGuire PK. Schizotypy and brain structure: a voxel-based morphometry study. Psychol Med. 2010;40: 1423–31. 10.1017/S0033291709991875 19917146

[47] Modinos G, Renken R, Ormel J, Aleman A. Self-reflection and the psychosis-prone brain: an fMRI study. Neuropsychology. 2011;25: 295–305. 10.1037/a0021747 21443341

[48] van der Meer L, De Vos AE, Stiekema APM, Pijnenborg GHM, Van Tol MJ, Nolen WA, et al Insight in schizophrenia: Involvement of self-reflection networks? Schizophr Bull. 2013;39: 1352–1362. 10.1093/schbul/sbs122 23104865 PMC3796073

[49] van der Meer L, Costafreda S, Aleman A, David AS. Self-reflection and the brain: A theoretical review and meta-analysis of neuroimaging studies with implications for schizophrenia. Neurosci Biobehav Rev. 2010;34: 935–946. 10.1016/j.neubiorev.2009.12.004 20015455

[50] Saxe R, Wexler A. Making sense of another mind: The role of the right temporo-parietal junction. Neuropsychologia. 2005;43: 1391–1399. 10.1016/j.neuropsychologia.2005.02.013 15936784

[51] Eddy CM. The junction between self and other? Temporo-parietal dysfunction in neuropsychiatry. Neuropsychologia. Elsevier; 2016;89: 465–477. 10.1016/j.neuropsychologia.2016.07.030 27457686

[52] Kriegeskorte N, Simmons WK, Bellgowan PSF, Baker CI. Circular analysis in systems neuroscience–the dangers of double dipping. Nat Neurosci. 2009;12: 535–540. 10.1038/nn.2303 19396166 PMC2841687

